# Genetic Diversity as Consequence of a Microaerobic and Neutrophilic Lifestyle

**DOI:** 10.1371/journal.ppat.1005626

**Published:** 2016-05-11

**Authors:** Nora-Johanna Krüger, Marie-Theres Knüver, Anna Zawilak-Pawlik, Bernd Appel, Kerstin Stingl

**Affiliations:** 1 Federal Institute for Risk Assessment, Department of Biological Safety, National Reference Laboratory for *Campylobacter*, Berlin, Germany; 2 Institute of Immunology and Experimental Therapy, Polish Academy of Sciences, Department of Microbiology, Wroclaw, Poland; University of California Davis School of Medicine, UNITED STATES

## Abstract

As a neutrophilic bacterium, *Helicobacter pylori* is growth deficient under extreme acidic conditions. The gastric pathogen is equipped with an acid survival kit, regulating urease activity by a pH-gated urea channel, opening below pH 6.5. After overcoming acid stress, the bacterium’s multiplication site is situated at the gastric mucosa with near neutral pH. The pathogen exhibits exceptional genetic variability, mainly due to its capability of natural transformation, termed competence. Using single cell analysis, we show here that competence is highly regulated in *H*. *pylori*. DNA uptake complex activity was reversibly shut down below pH 6.5. pH values above 6.5 opened a competence window, in which competence development was triggered by the combination of pH increase and oxidative stress. In contrast, addition of sublethal concentrations of the DNA-damaging agents ciprofloxacin or mitomycin C did not trigger competence development under our conditions. An oxygen-sensitive mutant lacking superoxide dismutase (*sodB*) displayed a higher competent fraction of cells than the wild type under comparable conditions. In addition, the *sodB* mutant was dependent on adenine for growth in broth and turned into non-cultivable coccoid forms in its absence, indicating that adenine had radical quenching capacity. Quantification of periplasmically located DNA in competent wild type cells revealed outstanding median imported DNA amounts of around 350 kb per cell within 10 min of import, with maximally a chromosomal equivalent (1.6 Mb) in individual cells, far exceeding previous amounts detected in other Gram-negative bacteria. We conclude that the pathogen’s high genetic diversity is a consequence of its enormous DNA uptake capacity, triggered by intrinsic and extrinsic oxidative stress once a neutral pH at the site of chronic host colonization allows competence development.

## Author Summary

Natural transformation, i.e. the capacity to take up DNA from the environment, is one of the crucial means for horizontal gene transfer and genetic diversity in bacteria. The human gastric pathogen *Helicobacter pylori* is confronted with acid stress before entering its multiplication site, the gastric mucosa. The bacterium causes lifelong chronic gastritis and is perfectly adapted to the human host, crucially by displaying unusual genetic diversity. Using a single cell approach and well-controlled conditions, we show here that the amount of imported DNA in competent *H*. *pylori* is outstanding, far exceeding previous measurement with other Gram-negative bacteria. Furthermore, DNA uptake activity was tightly regulated and limited to pH above 6.5, conditions thought to be met in close contact with the gastric mucosa. In addition, we show that within this pH competence window, competence development was triggered by an increase in pH in combination with the level of oxidative stress. Our data provide explanations for the extraordinary high genetic diversity, often referred to as genome plasticity of this unusual microaerobic pathogen.

## Introduction

The human stomach is a hostile niche. After overcoming the acid barrier, bacterial colonization at the gastric mucosa represents a constant battle with the host immune system. In particular, the pathogen is confronted with ROS-mediated oxidative stress produced by NADPH oxidases that release superoxide (O_2_^-^) [1]. Progression of disease towards carcinogenesis is also accompanied by DNA damage of host cells [2, 3]. *Helicobacter pylori* are genetically highly variable, microaerobic Gram-negative bacteria that successfully colonize the human gastric mucosa of half the world’s population, with variable prevalence in different human populations [4]. The bacterium causes lifelong gastritis and is also associated with mucosal-associated lymphoma and gastric adenocarcinoma [5]. *H*. *pylori* is a neutrophilic organism, but highly adapted to acid survival. For this purpose, the pathogen has a potent urease enzyme, which is tightly regulated via substrate accessibility by a pH-regulated channel [6, 7]. The microaerobic pathogen is also equipped with a set of oxidative stress enzymes, including catalase (KatA), alkyl hydroperoxide reductase (AhpC) and superoxide dismutase (SodB), which are important for host colonization [8–10]. *H*. *pylori* is not only exceptional regarding its colonization capacity of a harsh environment, but also regarding its enormous genetic diversity, crucially based on horizontal gene transfer and high recombination frequency [11]. In *H*. *pylori* horizontal gene transfer can be accomplished by natural transformation [12], i. e. the capacity to take up naked DNA from the environment. However, also direct exchange of DNA between *H*. *pylori* cells by conjugation-like mechanisms were shown to occur [13–15] and were discussed to play a yet underestimated role in genetic diversity of the gastric pathogen [16]. In contrast to all other known bacteria, DNA uptake in *H*. *pylori* during natural transformation is established by a type IV secretion system that is encoded by two separate operons, *comB2-B4* and *comB6-B10* [12, 17]. By tracking fluorescent DNA during uptake, we previously showed that DNA uptake in *H*. *pylori* is a two-step process [18]. The ComB system mediated transport of external dsDNA over the outer membrane into the periplasm. Our data also indicated that the conserved inner membrane localized ComEC channel, previously described by [19, 20] is involved in subsequent transport of probably ssDNA into the cytoplasm of *H*. *pylori* [18]. Monitoring fluorescent DNA during natural transformation was subsequently applied to other Gram-negative bacteria, like *Vibrio* and *Neisseria* [21, 22]. A maximum of 40 kb of imported DNA was detected in the bacterial periplasmic space of *Neisseria*, limited by the binding capacity of the periplasmic DNA-binding protein ComE (ComEA homologue) [21], which is absent in *H*. *pylori* [23].

In most bacteria, the capacity for natural transformation is tightly regulated, by timely restricting competence phase and/or by preferential uptake of DNA from related species. Quorum sensing for induction of competent state enables *Bacillus subtilis*, *Streptococcus pneumoniae* and *Vibrio cholerae* to increase the chance of import of DNA from siblings [24–26]. In *Streptococcus pneumoniae*, natural transformation was additionally triggered by antibiotics or DNA-damaging agents [27]. In *Vibrio* competence is also regulated by the presence of the natural habitat marker chitin and regulatory circuits appeared to be interconnected [28]. In *Neisseria* species-specific DNA uptake sequences [29] recognized by an outer membrane receptor [30] select for DNA of related bacteria. A DNA uptake sequence also facilitated DNA uptake in *Haemophilus influenza* [31], with variants of this sequence and core bases recently detailed by a next generation sequencing approach [32]. Furthermore, *H*. *influenzae* was suggested to restrict competence state to conditions of starvation [33].

A previous study implicated also sequence bias to occur in *H*. *pylori* natural transformation [34]–probably at the level of recombination. However, single cell analysis clearly showed that *H*. *pylori* did not discriminate between own and foreign DNA at the level of DNA uptake [18], which confirmed previous predictions of absence of redundant sequence motifs in the gastric pathogen [35]. In addition, natural transformation was long thought to occur constitutively in *H*. *pylori*. However, multiple competence phases and strain-dependent differences in natural transformation were described [36]. A study suggested that DNA damage exerted by the fluoroquinolone ciprofloxacin triggered natural transformation [37]. Interestingly, increased transformation rate was observed for *H*. *pylori* grown on agar plates incubated at slightly increased pH or different atmospheric composition [38].

Using a single cell approach, tracking fluorescent DNA in competent bacteria, we show here that *H*. *pylori* tightly regulates its competence state. A pH above 6.5 opened a window of opportunity, in which competence development was triggered by further increase in pH in combination with oxidative stress. Furthermore, single cell quantification of imported DNA provided evidence of a stunning DNA uptake activity, providing an explanation for the pathogen’s outstanding genetic diversity.

## Results

### Natural transformation is dynamically regulated in *H*. *pylori*

**Fig 1:**
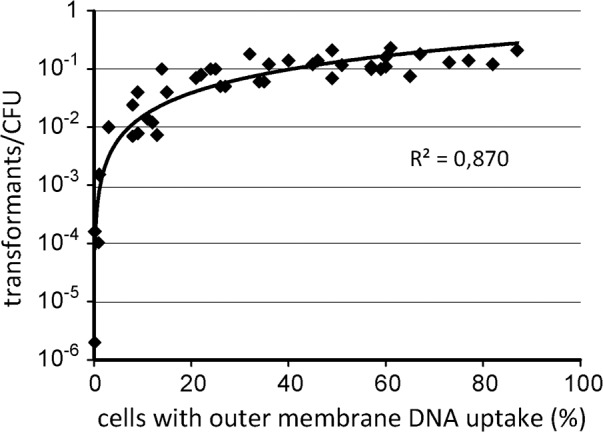
Transformation rate in *H*. *pylori* correlated with the fraction of cells with active outer membrane DNA transport. Cell suspensions were derived from the experiments illustrated in Fig 4. Bacteria with different competent fractions of cells were taken during or after competence development; non-competent cells were taken before competence development. *H*. *pylori* N6 were incubated in parallel with either Cy3-labelled λ DNA or a PCR fragment of *rpsL*(A128G), conferring streptomycin resistance. The data show the principle suitability of measuring the fraction of cells with active outer membrane DNA transport for the evaluation of competence development. Cumulative data from 13 independent experiments (n = 42).

In previous studies *H*. *pylori* competence was usually quantified by monitoring the expression of a resistance marker after contact with external DNA [36, 39]. This assay is not only dependent on the competent state but also on downstream effects like timely expression of the cassette and survival of the microaerobic bacterium under selection conditions. In addition to resistance marker expression, the transcription of a subset of genes essential for natural transformation was characterized in other studies [37, 38]. Here we directly monitor active DNA uptake by covalently labelling λ DNA with a Cy3 fluorophore and microscopically tracking DNA import in single cells. We showed previously that active DNA uptake of covalently modified DNA, such as Cy3-labelled λ DNA, occurred over the outer membrane at distinct locations and that DNA was trapped in the periplasm [18]. In this study, we wanted to use the assay for direct visualization of the competent state of *H*. *pylori*. Therefore, we first checked that the fraction of cells with incorporated fluorescent DNA, i. e. with at least one DNA focus, corresponding to active DNA uptake activity over the outer membrane, correlated with transformation rate. Thus, in parallel to the fluorescent assay counting cells with active DNA uptake activity, a second aliquot of the same cell suspension was incubated with a 1,022 kb DNA fragment harboring the *rpsL*(A128G) mutation for streptomycin resistance in order to measure natural transformation rate. We took cells suspensions from different growth phases before, during and after competence development as described below. As expected, the number of cells with one or more DNA foci correlated exponentially with the log number of transformants. Transformation rate ranged between 2 x 10^−6^ and 2 x 10^-1^ relative to total CFU (Fig 1). The lower limit of counted bacteria with active DNA transport was 0.2% (n ≥ 500 cells). When 10% cells with DNA foci were detected, transformation rate was around 1% (10^-2^). At a competent cell fraction of approximately 30% we observed a saturation of transformation rate of around 10^−1^ in *H*. *pylori* N6. As control and previously published for *H*. *pylori* strain J99 [18], N6 mutants with a defect in one of the essential proteins of the ComB system (*comB4* or *comB6*, [12, 17]) were impaired for natural transformation and displayed absence of active DNA uptake. These data reveal an enormous range of competence status in *H*. *pylori* spanning an order of log 5. The dynamics were controlled by variation of the amount of cells expressing active DNA uptake complexes. Hence, our assay of direct measurement of active DNA uptake was proven suitable for monitoring competence development.

Please note that highly transformable bacteria (transformation rate 10^−1^ to 10^−2^ tested using the streptomycin resistance point mutation marker) showed reduced transformation rate, when other resistance markers were used. When these cells were transformed in parallel with a chloramphenicol resistance cassette flanked by approximately 500 bp of homologous up- and downstream regions, 7 to 170-fold (median of 13-fold, n = 8) reduced transformation rates were observed. As expected, the transformation rate of the replicative vector pILL2157 [40] was log 3.8–6.4 less (median of log 5.9, n = 6) compared to the point mutation marker. Homologous recombination can occur in very short homologous regions [39]. Therefore, the chance for integrating a point mutation marker as compared to a complete resistance cassette is more likely, considering genetic barriers, in particular, restriction modification systems. A replicative plasmid has to reconstitute completely in the cytoplasm after single strand DNA uptake. Thus, the likeliness for full reconstitution of a plasmid is even less, compared to successful recombination of a resistance cassette with homology regions. Therefore, the data indicate that these highly transformable bacteria had substantial and to some extent variable genetic barriers that reduced the integrity of incoming DNA. These comprise, in particular, restriction modification systems, which were previously studied [41]. It also confirmed that the point mutation marker most faithfully detected DNA uptake.

### *H*. *pylori* competent cells import huge amounts of DNA within short time

**Fig 2:**
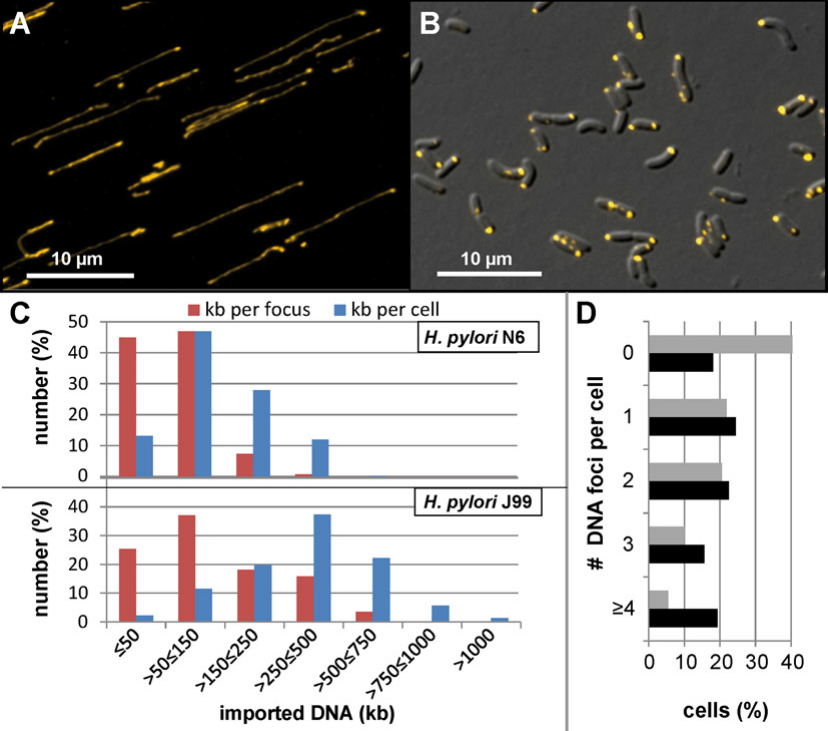
Quantification of imported DNA into *H*. *pylori* cells. A, Cy3 image of single Cy3-labelled λ DNA molecules, stained in yellow; B, DIC/Cy3 overlay image of *H*. *pylori* J99 after 10 min of DNA uptake under microaerobic conditions; C, results of the quantitative analysis of DNA uptake. For *H*. *pylori*, fluorescence intensity in single DNA foci (red) and in whole cells (blue) was determined in two different wild type strains (N6 and J99) after microaerobic growth in TSB-FBS into competent phase. D, histogram of the number of foci per cell. Grey bars, strain N6; black bars, strain J99. Fluorescence intensity was compared to that of single λ DNA molecules and expressed as imported kb. The images were acquired using a Zeiss Axio Observer Z1 and ZEN software, blue edition, version 2.0.0.0. n_cells_ = 928 or 1483 and n_foci_ = 1903 or 1740 were analyzed for J99 or N6, respectively.

For *H*. *pylori* the term “panmictic population structure” was used to express its enormous genetic variability [42]. We wondered if DNA import activity was also unusually high. For this purpose, we exploited the fact that *H*. *pylori* imports DNA independent of its source [18] and used DNA of the bacteriophage λ as standard for a 48.5 kb molecule. As mentioned above, we covalently labelled λ DNA with Cy3. In order to visualize the DNA molecules in a spread out way, a drop of DNA was added on an agarose pad and spread over the surface under concurrent air flow. Subsequently, the pad was sealed by a cover slip and mounted for microscopy. Under our experimental conditions, we expected that the chance for fully spread out λ DNA molecules is low. Hence, total fluorescence intensity of single Cy3 λ DNA molecules with a minimal length of ½ λ DNA (equals 8.6 μm, [43], Fig 2A) was determined from at least n = 120 molecules. This cut-off minimized the risk to quantify eventual breakage products and can maximally lead to a theoretical error of a factor two. However, the standard deviation of the measured fluorescence intensities was between 20.2% and 21.3%, confirming that we indeed measured mainly complete single molecules. Bleaching controls and linearity tests with different exposure times revealed high stability of the Cy3 fluorophore and guaranteed that bleaching was negligible under the experimental conditions (S1 Appendix, Fig A). Microaerobically grown competent *H*. *pylori* were challenged with the same lot of Cy3 λ DNA (1 μg/ml) in TSB-FBS for 10 min under microaerobic conditions at 37°C before DNaseI treatment for 5 min was performed (Fig 2B).

The amount of imported DNA was extraordinarily high in *H*. *pylori* cells. For strain J99, most of the cells had imported more than 250 kb (Fig 2C). We calculated a median value of 108 kb per DNA focus and 351 kb per cell. The maximum DNA amount per focus was 758 kb and 1.6 Mb per cell. Nearly 20% of all cells harbored 4 or more locally distinct DNA foci (Fig 2D). Hence, individual cells were capable of import of a chromosomal equivalent within short periods of time. The values for strain N6 were somewhat lower but still impressive. Here, most of the cells imported more than 50 kb, with a median of 124 kb. The median DNA amount in single foci was 55 kb. Maximum import of 392 kb per focus and 507 kb per cell were observed in strain N6. Thus, in contrast to other Gram-negative bacteria [21], the periplasm of *H*. *pylori* had a manifold higher maximal capacity for imported DNA. We analysed whether the DNA packaging density within single foci was also outstanding. Assuming that the fluorescence intensity in the analyzed regions of interest (ROIs) roughly correlated with the localization of DNA, we estimated the packaging density of periplasmic DNA by measuring DNA focus area. As a result, most of the imported DNA was packed in an area of 0.1–0.5 μm^2^ per λ molecule (S1 Appendix, Fig B). Minimally, we detected an area of 0.04 μm^2^ in J99. Assuming a spherical dimension of a DNA focus, this maximum package density corresponded to 0.006 μm^3^ per λ molecule, a DNA density of around 100-fold less than established in the icosahedron capsid of bacteriophage λ with an inner diameter of 55 nm [44].

### Imported DNA cannot complement purine deficiency

Since in other bacteria, DNA uptake was suggested to putatively play a role as nutrient supply, in particular in purine acquisition [45], we checked whether *H*. *pylori* competent bacteria can use external DNA as a purine source. For this purpose, *H*. *pylori* was grown in TSB-FBS and it was checked that at least 50% of the cells exhibited competence before the medium was exchanged for minimal medium without any purine source but with a supplementation of 10 μg/ml of DNA (*H*. *pylori* N6 genomic DNA or salmon sperm DNA). As control, growth in minimal medium was restored by addition of at least 5 μg/ml adenine (corresponding to 37 μM) and higher adenine levels (50 μg/ml) did not lead to enhanced growth (S1 Appendix, Fig C). However, when we added genomic DNA of *H*. *pylori* or of salmon sperm, the competent bacteria were not able to grow in minimal medium lacking adenine. A substantial fraction of bacteria was still capable to import DNA after 18–24 h of incubation in minimal medium as checked by transient detection of periplasmic DNA that was non-covalently stained by YOYO-1 and loss of signal within 60 min of incubation in the presence of external DNase (S1 Appendix, Fig D). Using a mutant capable of DNA import into the periplasm but deficient of DNA import into the cytoplasm (Δ*comEC*), the YOYO-1 signal had been shown to be rather stable in the periplasm of this mutant, thus, suggesting that complete loss of YOYO-1 signal in the wild type was due to import of DNA into the cytoplasm [18]. In addition, import of DNA into the cytoplasm was indirectly shown by transformability of the cells after incubation in minimal medium for 18–24 h using the *rpsL*(A128G) point mutation marker (transformation rate between 2 x 10^−2^ and 1.4 x 10^−1^). The results indicate that DNA was imported but could not complement for purine deficiency.

### Activity of DNA uptake complexes is reversibly shut down at slightly acidic pH

**Fig 3:**
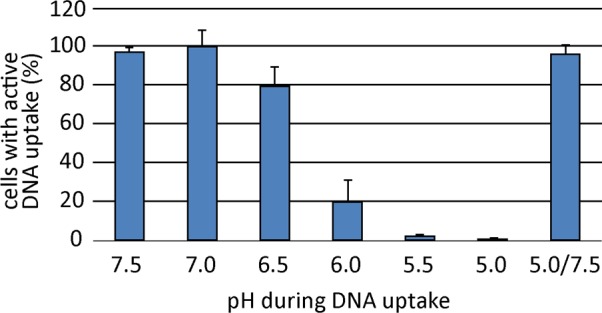
DNA uptake complexes are reversibly shut down at acidic pH values. Competent bacteria were incubated in BB-FBS that had been titrated with HCl or NaOH to the indicated pH values. Uptake of Cy3-labelled λ DNA occurred for 10 min under aerobic conditions. Cells were washed once in the respective pH medium before DNase treatment for 5 min in TSB-FBS at pH 7.5. The fraction of competent cells relative to the control condition incubated in TSB-FBS at pH 7.5 are depicted. Values stem from at least three experiments; error bars, standard deviation.

*H*. *pylori* encounters different pH values in its habitat, the human stomach. We tested the pH dependency of DNA uptake. The pH of BB-FBS was titrated at 37°C with HCl or NaOH to values between 5.0 and 7.5 in 0.5 unit steps and pH stability of these media was confirmed to be within ± 0.1 pH units. In order to keep the pH constant, the 10 min incubation was performed at 37°C under aerobic atmosphere, since higher CO_2_ concentrations used for microaerobic conditions led to pH decrease (see below). We started from a competent cell suspension exhibiting 63% ± 7.4% competent cells, which we defined as 100% (Fig 3). These values were obtained by incubation of the cells under our standard DNA uptake conditions in TBS-FBS at 37°C, except that the incubation was performed under aerobic conditions (when TSB-FBS exhibits pH 7.5). Changing the medium to BB-FBS with a pH of 7.5 demonstrated that medium did not influence the fraction of competent cells. As illustrated in Fig 3, the activity of DNA uptake was highly sensitive towards slightly acidic pH values. Below pH 6.5, DNA uptake complexes shut down activity and were practically inactive at pH 5.5. Interestingly, preincubation at pH 5.0 for 10 min and subsequent shift to pH 7.5 reactivated DNA uptake in *H*. *pylori* (Fig 3). Hence, we conclude that a neutral pH is a prerequisite of DNA uptake activity in *H*. *pylori*. In addition, the pH dependent regulation was tight, impairing DNA uptake at slight acidic conditions. Therefore, DNA uptake activity most likely occurs under neutral conditions, e.g. when *H*. *pylori* is in direct contact with the gastric epithelial cells.

### Neutral pH is a prerequisite of competence development triggered by oxidative stress

**Fig 4:**
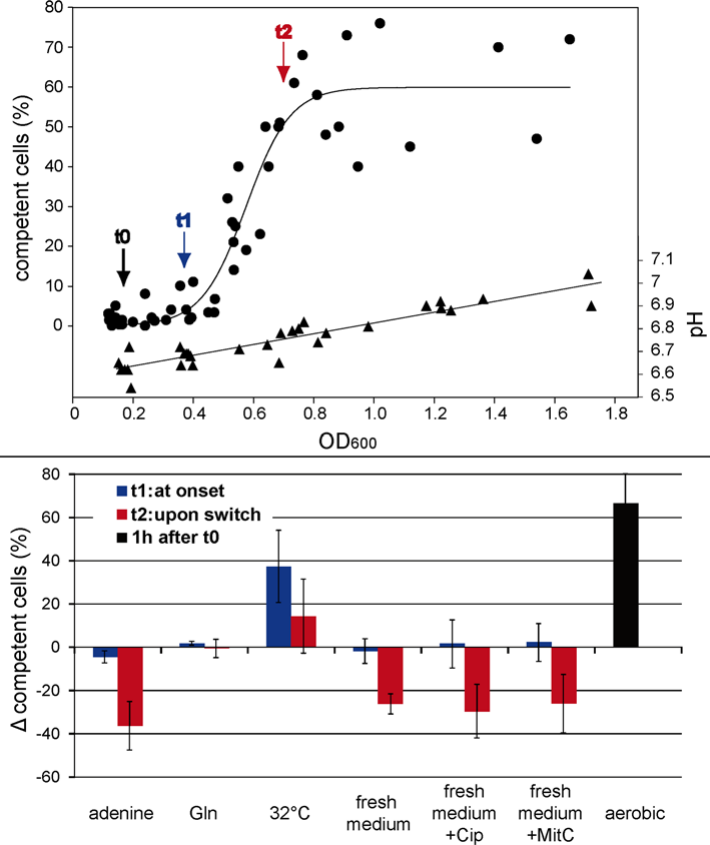
Competence development during microaerobic growth. Competence development was monitored by the fraction of cells with active outer membrane DNA uptake at distinct growth phases under microaerobic atmosphere at 37°C (n = 16; large graph, circles, with left y-axis, sigmoidal curve fit using Sigma Plot 11.0). pH was monitored during growth (large graph, triangles). *H*. *pylori* N6 was grown overnight in BB-FBS to an OD_600_ of 0.19 ± 0.05 (t0) at which only a minor fraction of cells displayed competence (2.2 ± 2.86%). The onset of competence development was defined at t1 at which cells exhibited a mean OD_600_ of 0.39 ± 0.05 and 4 ± 2.8% of competent cells (~ t0 + 3–4 hours). At t2 (~ t0 + 6–8 hours) upon switch into competent state (50.6 ± 15.6% of cells with active outer membrane DNA uptake) cells exhibited a mean OD_600_ of 0.7 ± 0.11. At t0, either effectors (0.5 mM adenine or 0.5 mM glutamine) were added or the medium was exchanged by fresh BB-FBS medium with our without supplementation of 0.125 μg/ml ciprofloxacin or 0.025 μg/ml mitomycin C or temperature was decreased for 5°C or the cell suspension was exposed to aerobic conditions. Differences in fraction of competent cells at t1 or t2 due to change in incubation conditions are shown in the inserted diagram (at least three experiments for each condition; error bars, standard deviation). For aerobic stress conditions data are depicted from time point 1h after t0.

We next monitored competence development, defined by the fraction of cells with active DNA uptake. Cells were grown microaerobically in BB-FBS overnight to an optical density of OD_600_ 0.19 ± 0.05 (t0), i. e. conditions of exponential growth phase. At this low OD_600_ *H*. *pylori* hardly exhibited any active DNA uptake complex (2.2 ± 2.86%). Between an optical density of OD_600_ 0.39 ± 0.05 (t1) and 0.7 ± 0.11 (t2), around 50% of the cells switched into the competent state (Fig 4). Concomitantly, the pH of the medium rose by 0.2 units during this incubation period (Fig 4).

In order to establish the parameters triggering competence development, we changed conditions at time point t0 and monitored alterations of the amount of the competent fraction at time points t1 and t2. Exchange of supernatant by fresh medium at t0 before the switch into the competent state reduced the fraction of competent cells at time point t2 (when the cells reached an OD of ~0.7, Fig 4, lower panel). Exchange of medium with fresh medium containing DNA-damaging compounds 0.125 μg/ml of ciprofloxacin or 0.025 μg/ml of mitomycin C, concentrations, which slowed down growth, did not lead to enhanced competence development. Instead, these conditions were similar to exchange with fresh medium without supplements (Fig 4, lower panel).

Interestingly, addition of 0.5 mM adenine at t0 inhibited the switch into competence state (competent fraction decrease of -36.3 ± 11.2% at t2), while the addition of 0.5 mM glutamine had no effect (Fig 4, lower part). Note that lower concentrations of adenine (0.05 mM), which were sufficient for purine complementation in minimal medium, had no effect on competence development (S1 Appendix, Fig E). This indicated that the effect of adenine on competence development was distinct from that of purine deficiency. A stimulating effect on competence development was observed by lowering incubation temperature by 5°C to non-optimal growth conditions (competence fraction increase of +37.2 ± 16.6% at t1 and +14.3 ± 17.3% at t2 relative to control cells). Compared to the control, the above mentioned conditions did not alter pH during incubation relative to the control. Exchange of medium by fresh BB-FBS accounted on average for -0.03 ± 0.03 pH units (maximum -0.07) at t2 relative to the control, while -0.01 ± 0.03 pH units deviation (maximum -0.05) were observed for addition of adenine and -0.07 ± 0.03 pH units (maximum -0.15) upon lowering the temperature for 5°C. These small pH deviations have unlikely caused the observed effects on competence development, in particular, because lowering the temperature by 5°C resulted in an increase rather than a decrease of the amount of competent cells at t2.

**Fig 5:**
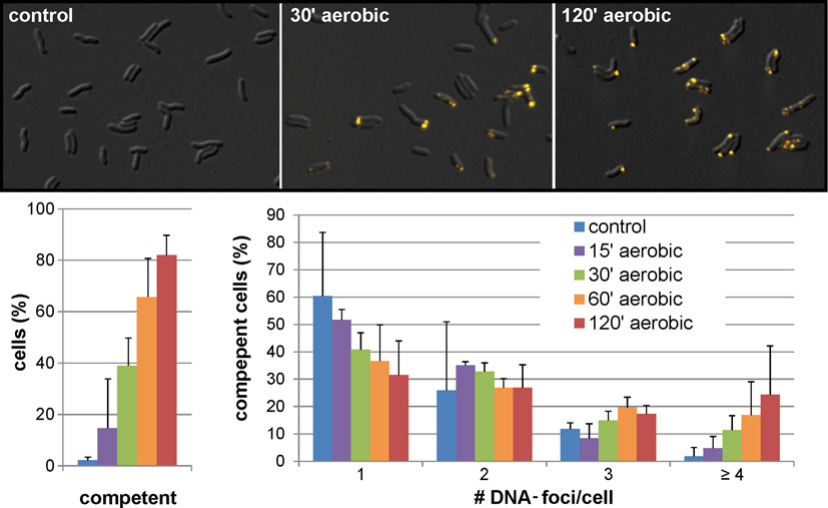
Kinetics of competence development under aerobic conditions. *H*. *pylori* N6 was grown microaerobically in BB-FBS overnight at t0 before competence development (OD_600_~0.2). Cells were exposed to aerobic conditions at 37°C for 120 min. At the indicated time points the competence fraction of the cells was monitored. Control cells were kept under microaerobic atmosphere. Upper panel, DIC/Cy3 images of bacteria at indicated timepoints, with DNA stained in yellow; lower panel (left), fraction of competent cells; lower panel (right), number of distinct DNA foci per competent cell. Data stem from at least three experiments; error bars, standard deviation.

The most pronounced effect on competence development was detected upon exposure to aerobic conditions (competent fraction increase of +67 ± 14% within the much shorter time period of 1 h compared to ~3–4 hours at time point t1 (Fig 4, lower panel, black bar)). Under the latter condition, we did observe a significant rise in pH, probably due to loss of CO_2_ from the medium upon switch from microaerobic to aerobic conditions (initial culture pH at t0 of 6.7 ± 0.06 and a final pH of 7.2 ± 0.06 after 1 h of aerobic incubation, see also below). In order to get an idea of the kinetics of competence development under aerobic conditions, samples were analyzed after 15, 30, 60 and 120 min (Fig 5), starting with a competent fraction of 2.2 ± 1.7%. *H*. *pylori* cannot grow under normal atmospheric conditions but survival was observed at least 2 h without loss of CFU (log CFU/ml at t0 was 8.2 ± 0.1 and at t2h 8.0 ± 0.7; n = 5). After 30 min of exposure to oxidative stress, already 38.9 ± 8.1% of the cells were capable of import of external DNA. After 60 min this fraction increased to 65.6 ± 14.8% and reached a maximum of 81.9 ± 7.9% after 120 min. We observed that upon 30 min of oxidative stress most of the competent cells exhibited more than one DNA focus, with over 20% of the competent cells harboring more than four visually distinct locations of active DNA uptake after 2 h of oxidative stress. This confirmed enormous DNA uptake capacity. Selected samples were measured for transformation rate, using the point mutation marker *rpsL*(A128G). We confirmed integration of the marker into the chromosome by recombination in agreement with the data in Fig 1.

**Fig 6:**
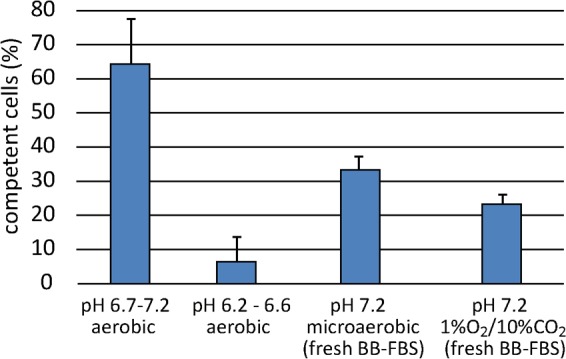
Neutral pH opens the opportunity for competence development triggered by oxidative stress. *H*. *pylori* N6 was grown microaerobically in BB-FBS overnight until growth phase before competence development (OD_600_~0.2). Cells were exposed to different pH and atmospheric conditions for 1 hour. Data stem from at least three experiments; error bars, standard deviation.

In order to dissect the role of pH and oxidative stress in triggering competence development, non-competent cells were exposed to aerobic conditions in BB-FBS for 1 h establishing various initial pH. As observed before, 64.3 ± 13.1% of the cells developed competence in non-titrated BB-FBS, with an initial culture pH at t0 of 6.7 ± 0.06 and a final pH of 7.2 ± 0.06 after 1 h of aerobic incubation (Fig 6). When the initial pH was titrated to 6.2 ± 0.09 before exposure to aerobic conditions and reached a final value of 6.6 ± 0.06 after 1 h, competence development was substantially inhibited (only 6.4 ± 7.5% competent cells). These results suggested that slight acidic pH prevented competence development. Furthermore, when we exchanged the medium with fresh BB-FBS titrated to an initial pH of 7.5 and incubated microaerobically for 1 h (corresponding to pH 7.2 ± 0.04 after equilibration of CO_2_), a significantly lower fraction of 33.3 ± 3.5% switched into the competence state compared to control cells under aerobic atmosphere within 1 hour (Fig 6). Note that the initial pH under microaerobic conditions was higher (pH 7.2 after CO_2_ equilibration) than in case of the aerobic condition (pH 6.7 rising to 7.2 during 1 h). From these data we conclude that competence development was not only pH-dependent but also triggered by the level of oxidative stress. Consistently, when oxidative stress was further reduced by using an atmosphere of 1% O_2_, 10% CO_2_ and 89% N_2_ and the same pH medium as for the microaerobic condition, the fraction of competent bacteria was further reduced (23.3 ± 2.5%, Fig 6).

Likewise, using TSB-FBS as a different growth medium, which had a slightly higher pH value (pH 6.8 ± 0.04 without *H*. *pylori* preincubation) than BB-FBS (pH 6.5 ± 0.04 without *H*. *pylori* preincubation), competence development under microaerobic conditions occurred earlier and was also inhibited by 0.5 mM adenine. At OD_600_ of 0.18 ± 0.05 (corresponding to t0) already 30.3% ± 15.1% of the cells were competent in TSB-FBS compared to 11.4% ± 8.3% in the presence of adenine (OD_600_ of 0.20 ± 0.07). At t1 (OD_600_ of 0.37 ± 0.05) the majority of bacteria (62.6% ± 6.2%) had switched into the competent state, whereas a reduced fraction of 37% ± 5.7% displayed competence when adenine was present (OD_600_ of 0.35 ± 0.03). Growth rates were comparable in both media.

A final experiment was conducted confirming that pH was a prerequisite of competence development and fine-tuned by the degree of oxidative stress. In this experiment, we titrated BB-FBS to distinct initial pH values (pH 6.6, 6.8, 6.9 and 7.0 after equilibration of CO_2_ under microaerobic conditions) and exchanged the medium of a non-competent cell suspension at t0 with these fresh media for further microaerobic incubation (S1 Appendix, Fig F). Note that the overall competence development under microaerobic conditions is slower than under aerobic conditions, confirming the results depicted in Fig 6. As observed for the control cells, the relative pH increased for about 0.2 units ± 0.05 during microaerobic growth over 6 hours. We observed that the increase of competent cells rose with increasing initial pH values (S1 Appendix, Fig F). For each pH condition, addition of 0.5 mM adenine inhibited competence development as observed before. The absolute effect of adenine inhibition was less with rising pH, putatively indicating that sensitivity towards oxidative stress might be increased with rising pH.

Taking together, the combined results are consistent with a competence window opening at a pH above 6.5. Subsequently, the combination of pH and oxidative stress appeared to define the kinetics of competence development in *H*. *pylori*.

### Growth of an oxygen-sensitive *H*. *pylori* mutant (*sodB*) is dependent on adenine

We further wanted to confirm that oxidative stress is indeed part of the trigger for competence development. Therefore, we intended to pinpoint the effect of adenine as radical quencher and constructed an oxygen-sensitive mutant of *H*. *pylori*. The oxidative stress enzyme superoxide dismutase (*sodB*) detoxifies superoxide radicals [46]. A mutant lacking SodB is confronted with higher oxidative stress because of oxygen radicals produced during microaerobic respiration. Likewise, *sodB* mutants of *H*. *pylori* were oxygen-sensitive and defective in mice colonization [10]. We constructed a mutant lacking SodB in *H*. *pylori* N6. Resuscitation of transformants at common microaerobic atmosphere (5% O_2_, 10% CO_2_, 85% N_2_) failed. However, transformants were obtained on Columbia blood agar at lower oxygen tension (1% O_2_, 10% CO_2,_ 89% N_2_). Also subculturing *sodB* mutants on Columbia blood agar under 5% oxygen were unsuccessful, suggesting that indeed the mutant exhibited an oxygen-sensitive phenotype.

**Fig 7:**
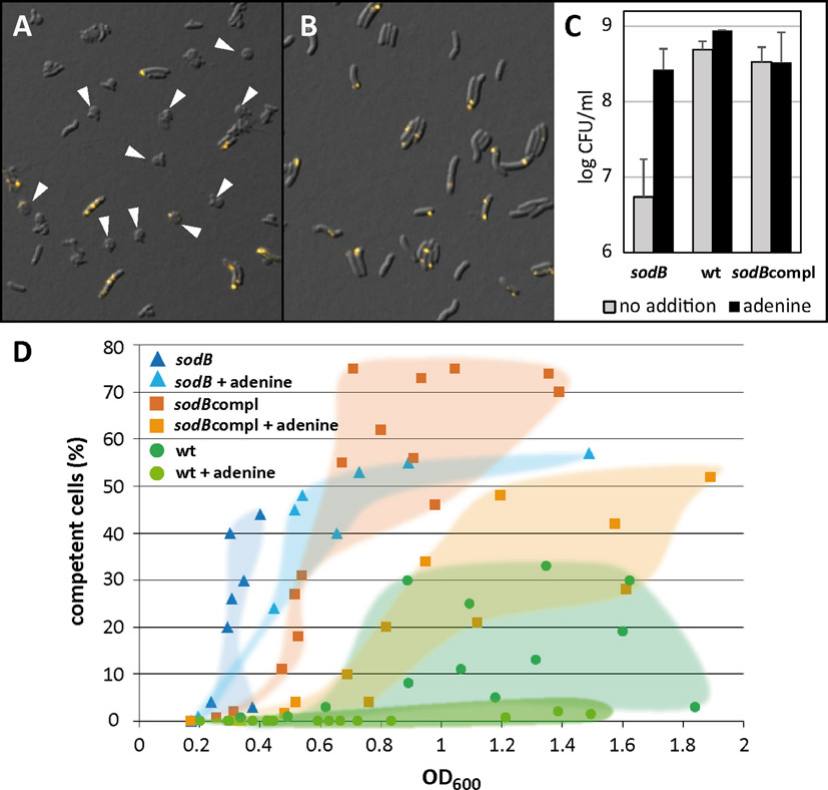
Phenotype of an oxygen-sensitive mutant *sodB* lacking superoxide dismutase. *H*. *pylori* were grown in BB-FBS at reduced oxygen atmosphere (1% O_2_, 10% CO_2_, 89% N_2_) in the absence and presence of 0.5 mM adenine. A and B, overlay images of DIC/Cy3 of *sodB* after 10 min of DNA uptake with DNA stained in yellow; arrowheads indicate coccoid formation of the *sodB* mutant in the absence of adenine (A), while cells kept their rod-shaped morphology in the presence of adenine (B). C, log CFU/ml after 18–24 h of growth of *sodB*, the wild type and the *sodB*compl. D, competence development occurred with lower OD_600_ values in the *sodB* mutant compared to the wild type; the *sodB*compl showed an intermediate phenotype. Addition of adenine reduced competence development. Data in C and D stem from at least three independent experiments; datapoints of the respective strain/condition were highlighted in D for better visualization.

In liquid culture (BB-FBS), where cells are dispersed and oxygen stress might be increased the mutant ceased to grow after some doublings and transformed into non-cultivable coccoid forms even at reduced oxygen level of 1% (Fig 7A). When we added 0.5 mM of adenine to BB-FBS, growth was partially complemented; the *sodB* mutant maintained its rod-shaped morphology and was re-cultivable (Fig 7B). After 18–24 h in BB-FBS at 1% oxygen, CFU were log 6.7 ± 0.5 in the absence of adenine and partially restored to 8.4 ± 0.3 in the presence of adenine (Fig 7C), which is consistent with the microscopic observation of coccoid formation in the absence of adenine. Complementation of the *sodB* deletion was performed by integration of *sodB* into the *fliP* locus (*sodB*compl). The *fliP* locus was previously used for insertion of a gentamycin cassette for the creation of a non-motile mutant and proven not to interfere with competence [18]. CFU of the wild type and the *sodB*compl in the absence or presence of adenine was similar ranging between log 8.5 and 8.9 (Fig 7C). Competence development occurred at lower OD values in the *sodB* mutant than in the wild type under similar conditions (Fig 7D). The *sodB*compl mutant showed an intermediate competence phenotype between the deletion mutant and the wild type, indicating that the deletion of *sodB* at its native locus was only partially complemented by insertion of *sodB* in the *fliP* locus. As shown before, addition of adenine resulted in a decrease of the competent fraction of cells. Note that consistently, the wild type also displayed lower competence development at 1% oxygen (Fig 7) as compared to 5% oxygen (Fig 4). The data support the following conclusions: (i) competence development during growth seemed to be triggered by oxidative stress and, (ii), adenine appeared to have an oxygen-protective role, probably as radical quencher.

Taking together, the data indicate that the opportunity for competence development was established at pH values above 6.5. Within this window, the combination of pH and oxidative stress triggered competence development. This might support the hypothesis that intrinsic (caused by respiration) as well as extrinsic oxidative stress (caused by oxygen tension and/or host inflammatory response) contribute to the impressive genetic diversity of the pathogen, colonizing close to the neutral gastric mucosa.

## Discussion

Using single cell analysis, we demonstrated that *H*. *pylori* cells import huge amounts of non-homologous DNA within short periods of time, the median amount ranging between 124 kb and 351 kb in two different strains tested (Fig 2). Individual cells were shown to import the equivalent of a complete genome (~1.6 Mb). This DNA amount far exceeded the previously quantified imported DNA in other Gram-negative bacteria, which was limited to 40 kb by the binding capacity of the periplasmic protein ComE (ComEA homologue) [21]. A homologue of *comEA* is missing in the *H*. *pylori* genome [23] and our data indicate an unknown alternative periplasmic protein with much higher binding capacity or higher abundance. However, compared to the natural packaging of λ DNA in the icosahedron capsid of the bacteriophage, DNA density in *H*. *pylori* periplasm was around 100-fold less. Yet the capsid of the bacteriophage λ spatially limits DNA packaging, resulting in high internal pressure from DNA bending, essential for DNA ejection [47]. The velocity of a DNA uptake complex in *H*. *pylori* was 1.26 kb/s [18], corresponding to a maximum import activity of 75.6 kb/min. We measured a maximum amount of 758 kb DNA per focus in J99 after 10 min of import. Although DNA uptake is processive in *H*. *pylori*, it is limited by the size of single λ DNA molecules and probably interrupted before binding and start of uptake of a new molecule. Therefore, it seems likely that a fluorescent DNA focus might also be the result of DNA import activity of multiple DNA uptake complexes in close proximity. This notion is corroborated by our previous observation of multiple concomitant uptake events into a single cell [18].

### Impact of oxidative stress on competence development

We observed that competence development occurred in liquid medium with increasing optical density. Exchange of the medium by fresh medium before the switch to the competent state inhibited competence development. A regulation by quorum sensing, however is not likely, as a similar effect was observed by adding adenine, conditions during which a putative autoinducer is not eliminated (Fig 4). In order to understand the effect of adenine (or fresh medium) on inhibition of competence development, we characterized a mutant deficient in superoxide dismutase, an important enzyme for oxidative stress defense [10]. The mutant was only able to grow at reduced oxygen levels. In BB-FBS, we observed that addition of adenine was crucial for prolonged growth and survival of the mutant, which turned into coccoids in its absence (Fig 7). Since superoxide dismutase is crucial for superoxide radical inactivation [46], this suggested that the effect of adenine on competence development in the wild type probably results from its radical quenching capacity [48]. Also, fresh medium might restore quenching capacity, which is depleted during growth. Note that the pH in the presence of adenine or after exchange of the medium by fresh medium at t0 was comparable to that of the control. Moreover, the *sodB* mutant displayed enhanced competence under similar conditions compared to the wild type (Fig 7), which supports the hypothesis that oxidative stress triggered competence development. The *sodB*compl mutant showed an intermediate competence phenotype between wild type and deletion mutant, indicating that the deletion of *sodB* at the native locus could only partially be complemented. Likewise, the most pronounced competence development was observed under aerobic conditions. Competence developed quite rapidly; within only 30 min of exposure, nearly 40% of the cells had switched into competent state and after 2 hours this fraction had increased to over 80%, with 20% of the competent cells expressing more than four distinct DNA uptake locations (Fig 5). Concomitantly, a pH elevation of around 0.5 units by loss of dissolved CO_2_ from the medium upon shift from microaerobic to aerobic atmosphere was observed, with implications discussed below.

We also tested other putative quencher molecules, like vitamin C and vitamin E. However, initial experiments showed that at those concentrations, at which competence development was inhibited, growth was drastically reduced, too. Therefore, these experiments were not followed up and data were not included in this manuscript, since substantial growth inhibition is expected to indifferently reduce protein biosynthesis. It might hint at the fact that quenchers can also impair the function of the respiratory chain. Instead, we showed by three independent experiments, (i) addition of adenine, (ii) variation of oxygen level and (iii) competence development in an oxygen-sensitive mutant that oxidative stress might play a crucial role in natural transformation.

#### Impact of pH on competence development

Transcriptome analysis previously indicated transcriptional repression of two essential genes for natural transformation (*comB8* and *comH*) at pH 5.4 relative to neutral growth conditions [49]. A recent study also hinted towards an effect of pH on natural transformation [38]. The authors conducted the experiments on agar plates, supplemented with a pH indicator. They could observe a 1- to 2-log increase in transformation rate, when the visually estimated pH was increased by about 0.2 units or when low CO_2_ concentrations were used. In our study we detected a comparable rise in pH by 0.2 units during growth in broth (Fig 4). Alteration of the culture conditions before switch into competent state and direct monitoring of single cell DNA uptake activity enabled us to gain more insight into the interplay of pH and oxidative stress and their effect on competence development.

The prerequisite of detection of fluorescent DNA taken up over the outer membrane is at least one fully active ComB-dependent DNA uptake complex per cell. ComB dependence was demonstrated by lack of DNA uptake activity in mutants harboring a deletion of one of the essential *comB* genes, either *comB4* or *comB6* [18]. The short-term DNA uptake assay (10 min of incubation in the presence of DNA) was performed at pH 6.8–7.5, a pH range at which we detected maximal DNA uptake activity of a competent cell suspension (Fig 3). Upon variation of pH during this short-time incubation, the majority of competent cells showed DNA uptake at pH above 6.5, while slight acidic pH did not lead to detectable DNA uptake. In addition, we were able to reactivate DNA uptake after short-time incubation at slight acidic pH values, indirectly demonstrating that the DNA uptake complexes were transiently inactive at pH below 6.5 (Fig 3). This led us to the conclusion that pH regulation took place at the level of DNA uptake activity (mediated by ComB transporter complexes).

However, pH regulation also occurred at the level of competence development. Starting with a practically non-competent cell suspension that did not show DNA uptake at DNA uptake assay conditions of pH 6.8–7.5 we monitored competence development under different incubation conditions over time (Figs 4–6). We conclude that the prerequisite for detection of DNA uptake activity over the outer membrane (at least one fully active DNA uptake complex per cell) was developed in response to pH alteration and oxidative stress in these experiments. Monitoring transcription of a subset of *comB* genes does not completely answer the question of whether the bacterium is fully equipped for DNA uptake. In contrast, visualization of DNA uptake activity during the experiment most directly shows competence of the bacteria, which is based on successful expression of all necessary genes (including *comB* genes), assembly of the proteins and eventual activation of the ComB transport system.

Does the tested pH range itself impact on chemical modification of DNA and, thereby, influence DNA uptake? Extreme acidic conditions might lead to some depurination, i. e. the release of purine bases from nucleic acids even at physiological salt concentrations and temperature [50]. The pH range used (pH 5-7.5) only covered slight acidic conditions. The cytoplasmic pH in *H*. *pylori* was approximately 5 at an external pH of 1 in the presence of urea [51], conditions under which survival of the bacterium was fully supported [52] and, thus, cytoplasmic DNA unaffected *in vivo*. At pH 5 DNA is expected to be more protonated compared to neutral conditions. Considering physiochemical properties, transport of a negatively charged macromolecule against the transmembrane potential (with negatively charged cytoplasm) might be facilitated at lower pH, at which net charge of DNA is reduced by protonation. However, our data show that under these pH values DNA uptake did not occur. Therefore, we conclude that biologically relevant pH regulation occurred, which might play a crucial role for the lifestyle of the gastric pathogen.

### Is DNA damage a trigger for competence development?

We showed competence development in BB-FBS under normal microaerobic growth conditions without external stress (Fig 4). From the overall data, we are tempted to conclude that (i) a slight increase in pH by approximately 0.2 units (above pH 6.5) in combination with (ii) decreasing quenching capacity of the medium led to competence development. It was suggested that the presence of antibiotics triggered natural transformation in *H*. *pylori* [37], as shown for *Streptococcus* and *Legionella* [27, 53]. In the study of Dorer and collegues the presence of ciprofloxacin resulted in a four-fold increase in transformation rate. This increase is rather marginal, considering dynamics of log 5 demonstrated in this study or around log 2 in the study of Moore et al. [38]. We tested ciprofloxacin and also mitomycin C under our atmosphere-, pH-, temperature- and growth phase controlled conditions. Fluoroquinoles are thought to generate double strand breaks in DNA [54, 55]. Mitomycin C crosslinks to DNA and causes DNA damage [56, 57]. We chose sublethal concentrations that slow down growth. Additionally, we tested higher concentrations and confirmed lack of competence induction as shown previously [37]. However, sublethal intermediate concentrations of either ciprofloxacin or mitomycin C also did not show any impact on competence development under our conditions (Fig 4). In contrast, our results demonstrated that small variations in atmosphere, external pH and temperature largely influence competence development. This might be an important reason for the relatively high variation in published transformation rates in different laboratories using various media and gas-generation systems. Furthermore, alterations of these parameters during processing of the sample within the experimental setup, including a drop of temperature have to be taken into account in order to prevent biased results.

### Identification of a pH-defined competence window in *H*. *pylori*

In *B*. *subtilis* the term “competence window” was used to define a time period, during which the likelihood for switching into competence state is increased by rising the basal level of the master competence regulator [58, 59]. Our data suggest that in *H*. *pylori* a “competence window” is opened and closed by external pH. In case the pH reached near neutral values (> pH 6.5), competence development was triggered by the combination of pH increase and oxidative stress. The kinetics of competence development was modifiable by oxygen tension (Fig 6) or by the addition of the radical quencher adenine (Fig 4) under comparable pH conditions. Drop in temperature to non-optimal growth conditions also led to increase in competence development, confirming that competence development was not purely regulated by external pH. The temperature effect might be explained by an imbalance of metabolic processes, putatively also leading to sub-optimal (oxidative?) stress defense.

From our data we conclude that DNA uptake in *H*. *pylori* is dispensable and eventually even deleterious under acidic conditions. In contrast, the process appears to be limited to neutral pH values. From transcriptome data it was suggested that *H*. *pylori* colonizes an acidic niche [60]. However, that study focused on early colonization (5–10 days after infection) of *H*. *pylori* in gerbils and not only surface epithelial cells with attached bacteria but also gastric mucus was collected as pool samples. Since the majority of *H*. *pylori* is thought to reside in the mucus layer, where slight acidic pH values are likely to occur, detection of acid induced genes may be explainable. However, these data do not necessarily reflect pH in direct contact with the host. Measurements using pH microelectrodes showed that *H*. *pylori* is confronted with a pH gradient lining from the near neutral gastric mucosa to the extreme acidic pH in the stomach lumen [61, 62]. Therefore, our data suggest that DNA uptake is most active in direct contact with the host, a site with near neutral pH. In early stages of *H*. *pylori* gastritis, the bacterium is preferentially localized at the antrum; with continued inflammation, gastrin producing cells are lost in the antrum, probably leading to decrease in acid secretion by the parietal cells and spread of bacteria and inflammation to the corpus [63]. Likewise, the pH value of the gastric mucosa in patients with ulcers was higher than for a control group [64]. At the sites of chronic severe inflammation, increased oxidative stress due to ROS production by the host might be expected [65].

We observed that, in contrast to *B*. *subtilis*, the majority of the *H*. *pylori* population acquired the competent state and growth of the bacterium was not interrupted. In *B*. *subtilis* natural transformation probably enables the bacterium to survive and adapt to changing unfavorable conditions. In this respect, transiently interrupting growth might serve for increased survival and enhanced persistence. Our data suggest that natural transformation in *H*. *pylori* is established under the most favorable conditions within the hostile stomach, namely at the multiplication site close to the near neutral mucosa. Triggering chronic gastritis, ROS-production by the host cell inflammatory system will be a daily reality for the gastric pathogen.

#### Role/s of natural transformation in *H*. *pylori*

What is the purpose of this high energy hungry process? Natural transformation was suggested to play a role in nutrient supply in other bacteria [45]. However, when we exposed highly competent *H*. *pylori* with external DNA in a minimal medium without purine source, growth was not supported (S1 Appendix, Fig C). Intriguingly, the bacterium was capable of import of DNA into the cytoplasm under these conditions (S1 Appendix, Fig D) but it was not able to recycle nucleotides for replication. Interestingly, *H*. *pylori* did not discriminate between own and foreign DNA, as demonstrated by the import of λ DNA ([18] and Fig 2). In principle, there are two explanations: First, there is no need to select for its own DNA in an ecological niche where DNA of siblings prevails [66]. Abundant human DNA will probably exert a limited risk for deleterious, non-homologous transformation events. By demonstrating that active DNA complexes were completely shut down at a pH ≤ 5.5, it is likely that DNA uptake is prevented in the acidic lumen, where the presence of DNA of foodborne bacteria is expected. In this scenario, a high level of DNA uptake from siblings can serve for DNA repair under oxygen stress conditions and/or for exchange of genetic material. The immune system exerts selection pressure on *H*. *pylori*, for which an environmental niche or other host reservoirs besides humans are not known. DNA repair systems were shown to be crucial for optimal colonization at the gastric mucosa and it was concluded that pathogen DNA is a target for host-generated oxidative stress [67]. Extensive horizontal gene transfer probably provides high adaptive capacity to create *H*. *pylori* diversity for enhanced fitness, in particular, for novel host infection.

Second and highly recommended to be considered, DNA uptake might serve a purpose beyond DNA repair and genetic diversification. We could imagine that the import of huge amounts of non-homologous DNA might establish a dilution effect. In this scenario, imported DNA might create a reservoir of oxidizable nucleotides, protecting own cytoplasmic chromosomal DNA against oxidative stress, in particular during host immune response. In this regard, genetic diversity would be a side product of oxidative stress defence. However, while genetic diversification was shown to be extraordinary in *H*. *pylori*, leading to a panmictic population structure, the latter putative hypothesis will require further analysis.

The *in vivo* relevance of the *comB* system for colonization was clearly demonstrated for *comB4* in the gerbil model [68]. However, since a *H*. *pylori* mutant, defective in the *comB8-B10* operon was only attenuated but not excluded from the gerbil stomach, the conclusion was that natural transformation was not essential for initial (3-weeks) colonization. Active chronic gastritis was detected at 3 to 8 weeks after infection in Mongolian gerbils [69–71], whereas ulcers were observed after approximately 6 months [70, 72] and gastric cancer after 18 months of infection [72]. In another study, a *comB10* mutant colonized mice during one week like the wild type, but colonization was somewhat decreased after 8 weeks in a competition experiment [73]. Interestingly, a *dprA* mutant, intact for DNA uptake but impaired in recombination of the DNA into the chromosome, did not show a defect in colonization after 8 weeks but displayed reduction after 12 weeks. A future systematic investigation of essential functions of natural transformation (outer membrane transport, inner membrane transport and recombination) in long-term colonization experiments—most appropriate in the gerbil model with human-like inflammatory response—will provide more insight into the multiple roles of natural transformation.

### Conclusions

In conclusion, we showed for the first time that during microaerobic growth neutral pH values defined a competence window in which DNA uptake capacity was triggered by a combination of pH and oxidative stress. In consequence, this led to the import of staggering amounts of DNA into the majority of cells within the population. Concomitantly, we detected high transformation rates under these conditions. Hence, our data deliver explanations for the observed extraordinary genetic diversity of this microaerobic pathogen, persisting lifelong at a near neutral multiplication site under conditions of chronic inflammation.

## Materials and Methods

### Strains and growth conditions

*H*. *pylori* strains N6 [74] and J99 [75] were grown either on Columbia blood agar base (Oxoid) supplemented with 5% defibrinated sheep blood (Oxoid, Heidelberg, Germany) or in liquid culture (shaking at 140 rpm) using tryptic soy broth (TSB, Becton Dickinson, USA) or brucella broth (BB, Becton Dickinson) with 5% fetal calf serum (PAN-Biotech GmbH, Aidenbach, Germany), in the following referred to as TSB-FBS or BB-FBS, respectively. Antibiotics were used at the following final concentrations per milliliter: 12.5 μg vancomycin, 0.31 μg polymyxin B, 6.25 μg trimethoprim, and 2.5 μg amphotericin B. If applicable, kanamycin or chloramphenicol was added at 20 μg/mL or 8 μg/ml, respectively. Plates were incubated at 37°C in a microaerobic incubator (Binder, Tuttlingen, Germany) at 1% or 5% O_2_, 10% CO_2_ and 85% N_2_. Atmosphere for liquid cultures was established in jars by evacuating air and refilling the volume with defined gas mixture (1% or 5% O_2_, 10% CO_2_, rest N_2_).

The minimal medium was composed according to [76] but lacked the sole purine source adenine, which was added at concentrations of 5 or 50 μg/ml (corresponding to 0.037 or 0.37 mM, respectively). Instead of addition of single amino acids, 100 x MEM non-essential amino acids solution (Life Technologies) was used as a 20-fold stock solution and 50 x MEM amino acids solution (Life Technologies) as a 10-fold stock solution, matching the amino acid concentrations of the original minimal medium except for 66 mg/l asparagine, 210 mg/l histidine and 180 mg/l tyrosine. Glutamine was added separately according to the original protocol. The pH of the medium was adjusted to 7.4. For supplementation of the medium with 10 μg/ml DNA, either DNA of salmon sperm (Sigma Aldrich) or purified chromosomal *H*. *pylori* N6 DNA was used.

### *H*. *pylori* mutants used in this study

Oligonucleotides used in this study are depicted in S1 Appendix, Tab A. Genomic DNA of a streptomycin resistant mutant of *H*. *pylori* strain 26695, bearing an A128G mutation in *rpsL* was used as template for the amplification of a 1,022-bp *rpsL*(A128G) PCR fragment using oligos H3 and H6. N6 *comB4* and N6 *comB6* were constructed by transformation of a PCR fragment amplified on genomic DNA from the respective J99 mutants published previously [18] and selected on 20 μg/ml kanamycin.

Approximately 500-bp flanking regions of *sodB* (HP0389; ORF definition of 26695) were amplified using oligos H167/H168 (5′ region including start codon of *sodB*) and H169/H170 (3′ region including 39 bp of *sodB*) and genomic DNA of N6 as template [77]. The nonpolar kanamycin resistance cassette was amplified from pUC18ΔK2 [78] using oligos H13 and H14. Since the oligo H168 contains 22 bp, which are reverse complementary to H13, and H169 contains 21 bp, which are reverse complementary to H14, the nonpolar kanamycin cassette was inserted between the flanking regions by fusion PCR. This led to a fusion PCR fragment, suitable for nonpolar inactivation of *sodB* in N6 by direct transformation and selection on 20 μg/mL kanamycin. For complementation of the *sodB* mutant, a 1.2 kb fragment comprising *sodB* including the annotated transcriptional start site [79] and approximately 500 bp 5’ region was amplified using H167 and H177 and genomic DNA of N6 as template. The gentamicin cassette was amplified using H11 and H12 from pUC1813*apra* [80]. Approximately 600-bp flanking regions of the insertion locus *fliP* were amplified using oligos H7/H178 (5’ region) and H9/H10 (3’ region). Since the oligo H178 contains a 20 bp reverse complementary region to H167, the oligo H177 a 21 bp reverse complementary region to H11 and the oligo H9 a 22 bp reverse complementary region to H12, all four fragments were fused by PCR using oligos H7 and H10, resulting in an approximately 3.2 kb fragment. Correct allelic exchange of all mutants was checked by PCR and sequencing.

### Fluorescence labeling of DNA and bacterial uptake

λ DNA (*dam*- and *dcm*-; Thermo Fisher Scientific, USA) was covalently fluorescently labeled applying Mirus Label IT Cy3 (MoBiTec GmbH, Goettingen, Germany) according to the manufacturer’s protocol using a 1:1 (volume: weight) ratio of Label IT reagent to nucleic acid.

λ DNA was non-covalently labeled with the fluorescent dye YOYO-1 (Invitrogen, USA) at a bp:dye ratio of 1:50 in phosphate-buffered saline a pH 7.2. YOYO-1 specifically stains dsDNA by intercalation at bp:dye ratios of >8 and is virtually non-fluorescent in aqueous solutions [81]. One microgram per ml of labeled DNA was added to a *H*. *pylori* cell suspension, which had been centrifuged and resuspended in TSB-FBS (100 μl OD_600_ ∼0.2–0.8). After incubation for 10 min at 37°C under microaerobic atmosphere, the suspension was centrifuged, resuspended in 25–50 μl TSB-FBS and incubated for 5 min at 37°C in the presence of 10 units of DNaseI (Roche, Basel, Switzerland).

### Quantification of imported DNA

For quantification of fluorescence intensity from single λ DNA molecules, a drop of freshly labelled Cy3 λ DNA diluted in TSB was spread out on a 1.5% low melting agarose pad by air flow and immediately sealed with a cover slip. In parallel, *H*. *pylori* cells were incubated for 10 min in the presence of the same lot of Cy3 λ DNA. Fluorescence microscopy was performed using a Zeiss Axio Observer Z1 microscope with a plan apochromatic 63x/1.4 objective and differential interference contrast (DIC). Cy3 was visualised using a metal halide light source (HXP120C) and a filter set with excitation at 550 nm (bandwidth 25 nm), emission at 605 nm (bandwidth 70 nm) and a dichroic beamsplitter at 570 nm. YOYO-1 that intercalated into dsDNA was detected with a filter set providing excitation at 470 ± 20 nm and emission at 525 ± 25 nm (dichroic beamsplitter at 495 nm). Images were acquired with the 12-bit monochromatic AxioCam MRm camera, using exposure times between 60 ms and 2.5 s. Intensity values of Cy3 were checked for linearity between different exposure times and bleaching was found negligible (S1 Appendix, Fig A). Regions of interest (ROIs) were set manually and analyzed by ZEN software (blue edition) version 2.0.0.0. In order to determine the fluorescence of one single λ DNA molecule, fluorescence intensity was determined from spread out DNA molecules with a minimal length of ½ of maximal size of λ DNA (8.6 μm, [43]) and defined as one molecule. Fluorescence intensity in single DNA foci and in whole cells were determined, compared to fluorescence intensity of λ DNA molecules and expressed as imported kb. The cut-off value for the analysis of DNA foci was set to a signal to background ratio of ≥ 2.

### Determination of transformation rate

For the determination of transformation rate, bacterial cells grown in liquid culture were centrifuged and resuspended in 100 μl TSB-FBS to OD_600_ ∼1. Subsequently, 1 μg/ml of a 1,022-bp *rpsL*(A128G) PCR fragment was added and incubated for 1 h at 37°C under microaerobic atmosphere. Cells were centrifuged, resuspended in 50 μl TSB-FBS in the presence of 10 U of DNaseI (Roche, Basel, Switzerland) and directly spotted on Columbia blood agar. After incubation under microaerobic conditions at 37°C for 20 ± 4 h, bacteria were harvested in TSB-FBS. Total colony forming units as well as the number of streptomycin resistant transformants were determined according to the serial dilution method using Columbia blood agar with and without 20 μg/mL streptomycin and a selection time of 4–5 days. As alternative resistance marker, the chloramphenicol cassette of pILL2157 [40] was used within the context of this replicative plasmid or amplified using oligos H108 and H109 and fused to approximately 500 bp 5’ region (oligos H110 and H111) and 3’ region (oligos H112 and H113) of *nucT* in order to create a 1886 bp PCR fusion fragment (H110/H113) for homologous recombination into the *nucT* locus.

### Acceleration/deceleration of foci development

Cells were inoculated at OD_600_ ~0.003 and grown over night under microaerobic conditions in BB-FBS to time point t0, at which the culture had reached approximately OD_600_ between 0.15 and 0.25. At t0 either one of the different conditions was established; (i) one of the effectors (see below) was added or (ii), the culture was temperature downshifted by 5°C or (iii), the culture was exposed to aerobic conditions at 37°C or (iv), the medium was exchanged for fresh medium with or without supplementation of either one of the DNA-damaging agents 0.125 μg/ml ciprofloxacin or 0.025 μg/ml mitomycin C. It was guaranteed that the procedure for changing the condition was completed within 10 min. For exchanging the medium, cells were centrifuged at 9000 x g for 2 min at room temperature and immediately resuspended in the same volume of prewarmed (37°C) fresh medium with or without DNA-damaging agents. At t1 (3–4 h after t0) and t2 (6–8 h after t0) a test aliquot was taken from the cultures and checked for the number of cells harboring DNA foci as mentioned above. For aerobic exposure, samples were taken after 15, 30, 60 and 120 min, respectively. If indicated, pH was measured using a temperature-controlled pH electrode with an accuracy of 0.01 pH units (pH/ATC, Denver Instrument, New York, USA). In order not to disturb competence development, parallel culture aliquots served for pH measurements.

## Supporting Information

S1 Appendix Includes Supplementary Table A and Supplementary Figures A-F. Table A: Oligonucleotides used in this study. Figure A: Bleaching curve of Cy3 labelled λ DNA. Figure B: Distribution of estimated area per λ molecule in periplasmic DNA foci of *H*. *pylori*. Figure C: Growth of competent *H*. *pylori* N6 in minimal medium without purine source cannot be restored by addition of external DNA. Figure D: Competent *H*. *pylori* did not grow in minimal medium in the absence of a purine source but were still capable of DNA uptake. Figure E: Competence development is not influenced by lower concentrations of adenine. Figure F: pH is a prerequisite of competence development and fine-tuned by the degree of oxidative stress.
(PDF)
