# Supplementary material for: Genetic Diversity as Consequence of a Microaerobic and Neutrophilic Lifestyle
Source: PLoS Pathog. 2016 May 11;12(5):e1005626. doi: 10.1371/journal.ppat.1005626 (PMC4864210; doi:10.1371/journal.ppat.1005626)
Supplement: S1 Appendix — Table A: Oligonucleotides used in this study. Figure A: Bleaching curve of Cy3 labelled λ DNA. Figure B: Distribution of estimated area per λ molecule in periplasmic DNA foci of H. pylori. Figure C: Growth of competent H. pylori N6 in minimal medium without purine source cannot be restored by addition of external DNA. Figure D: Competent H. pylori did not grow in minimal medium in the absence of a purine source but were still capable of DNA uptake. Figure E: Competence development is not influenced by lower concentrations of adenine. Figure F: pH is a prerequisite of competence development and fine-tuned by the degree of oxidative stress. (PDF) [file ppat.1005626.s001.pdf]

S1 Appendix: Supporting Information

Table A. Oligonucleotides used in this study

Name	5'-3' sequence	reference
H3	CGTGTGATCGCTTTAAAAGGCG	[18]
H6	CCACTCTTCTGCTGCGCACTTC	[18]
H7	ACGCGCCCATAACCTTTAGGG	[18]
H9	CGGAGAACGAGATGACGTTGGAATGGGTATGATGATGCTCCCGC	[18]
H10	GGAAGCGCTTGCCCTATGTGAG	[18]
H11	GCAGCTCCATCAGCAAAAGGG	this study
H12	TCCAACGTCATCTCGTTCTCCG	this study
H13	GCTCGGTACCCGGGTGACTAAC	[18]
H14	TCCCCGGGTCATTATTCCCTC	[18]
H15	GTGCGTTTCAAGTTTGTTCGCTG	[18]
H18	CCCCTAAAGAAGTCTTATAATCCCTTGC	[18]
H19	GTTTGGTGGTAGCGCAATTTGATC	[18]
H22	ATAATGGCGTAATGCTTGTCCTGG	[18]
H108	CAACAGATCCGAGATTTTCAGG	this study
H109	GTTTAAGGGCACCAATAACTGC	this study
H110	CTTGCGTTAGAGCCAAGC	this study
H111	CCTGAAAATCTCGGATCTGTTGGCTTGCAAAACCCCTAAAC	this study
H112	GCAGTTATTGGTGCCCTTAAACGATGCTAGAAGGTTGCGTTG	this study
H113	CGGTTACACCAATGTAATGACTAG	this study
H167	GCCACAAATTTGTCCTTGAG	this study
H168	GGTGATATTCTCATTTTAGCCATGTTTTCTCCTTGTGATCAG	this study
H169	TTTTAGTACCTGGAGGGAATAATGGTGGATTACTACATCAATGAGTTGG	this study
H170	CAACGCAAGGCTGTTATAAATC	this study
H177	CCCTTTTGCTGATGGAGCTGCCCTTTAAACCACTCTATGCAAAATAACGC	this study
H178	CTCAAGGACAAATTTGTGGCGCGCTATCCGCGCTCATTAAG	this study

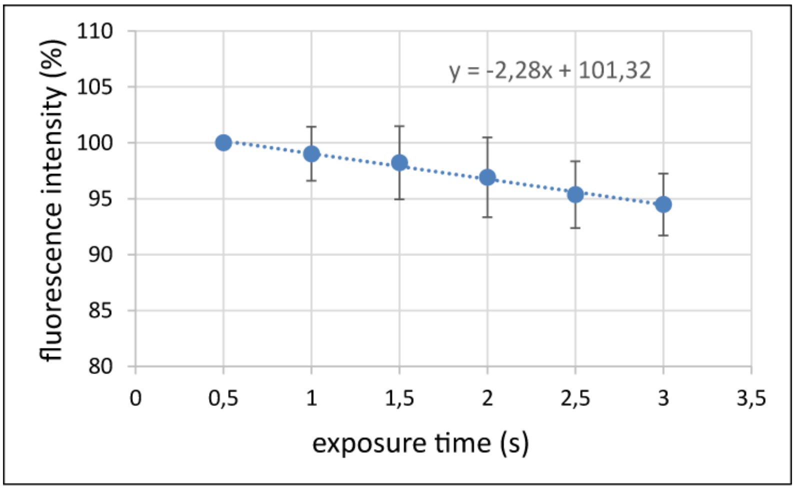

Figure A. Bleaching curve of Cy3 labelled λ DNA. Multiple consecutive images were taken from Cy3 labelled λ DNA with exposure times between 500 ms and 2 s and the fluorescence intensity of distinct ROIs was determined along the bleaching process. Fluorescence intensity of the first image was set to 100 %. Cumulative data are shown from three experiments (n ≥ 140). Fluorescence intensity decreased about 2.3 % per second of exposure.

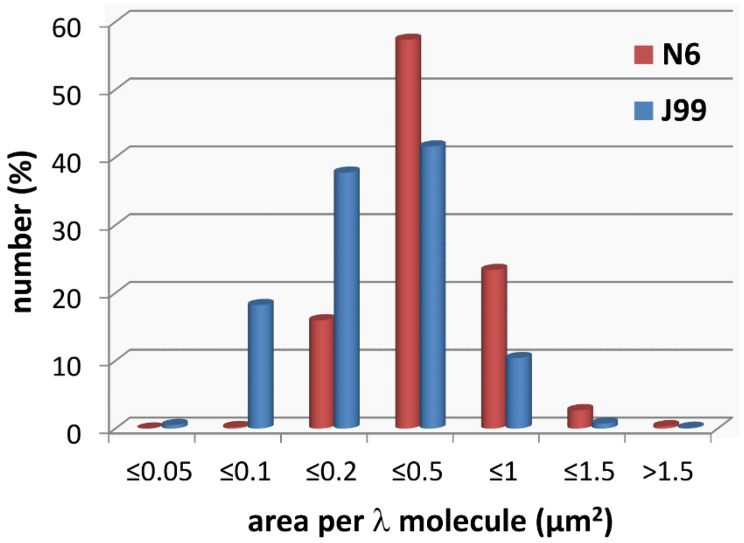

Figure B. Distribution of estimated area per λ molecule in periplasmic DNA foci of *H. pylori*. Assuming that the fluorescence signal in the analyzed ROIs (data of Fig. 2) roughly correlated with the localization of DNA, we estimated focus area and area per λ DNA molecule packed in a DNA focus.

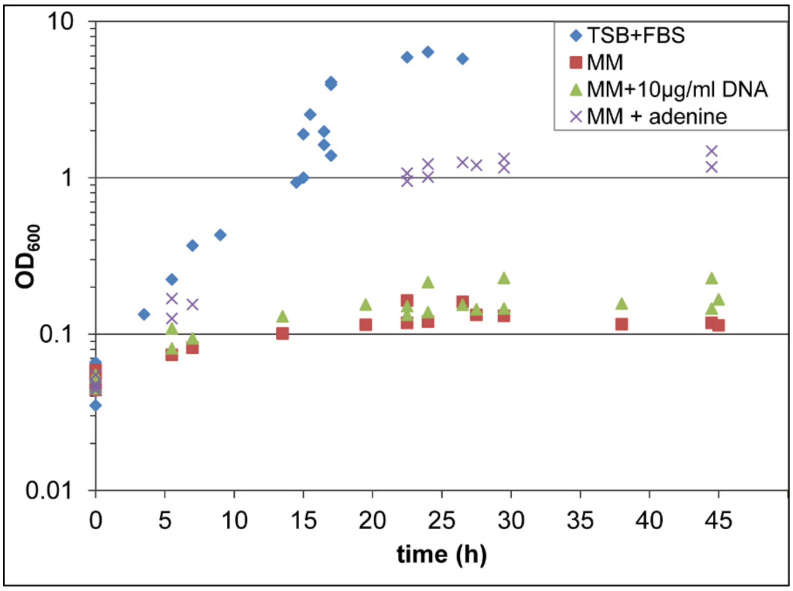

Figure C. Growth of competent *H. pylori* N6 in minimal medium without purine source cannot be restored by addition of external DNA. When indicated, either adenine (cumulative data of addition of 5 or 50 µg/ml) or DNA (cumulative data of addition of 10 µg/ml salmon sperm DNA or 10 µg/ml purified N6 chromosomal DNA) was added. As control, cells were grown in TSB-FBS. Cumulative data from at least four growth experiments.

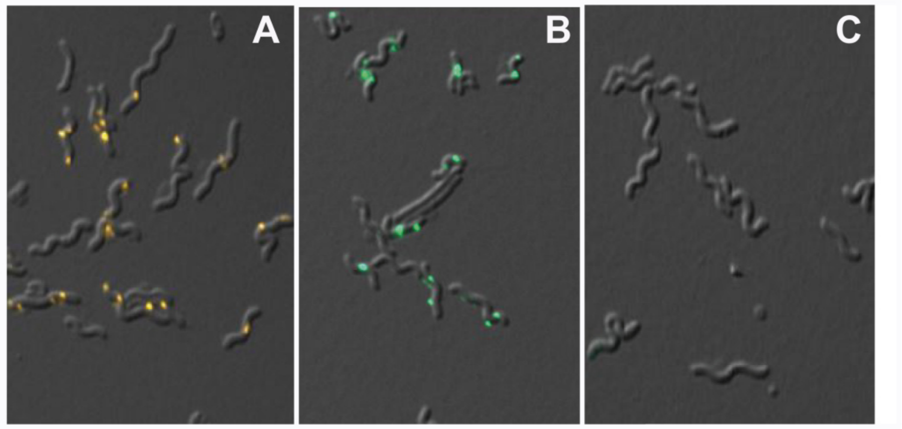

Figure D. Competent *H. pylori* did not grow in minimal medium in the absence of a purine source but were still capable of DNA uptake. N6 wild type cells were challenged with fluorescently labelled λ DNA after 18-24 h of incubation in miminal medium (see also Fig S1). A, Cy3/DIC overlay image, with DNA stained in yellow; bacteria imported covalently labelled Cy3 λ DNA into the periplasm, which is periplasmically trapped. B, YOYO-1/DIC overlay image, with DNA stained in green; bacteria imported non-covalently labelled YOYO-1 λ DNA into the periplasm; image was taken after 10 min of import and 5 min of DNase treatment (t0). C, bacteria in B were further incubated microaerobically for 60 min at 37°C, during which YOYO-1 labelled λ DNA had been transported into the cytoplasm and caused loss of YOYO-1 signal as previously demonstrated [18].

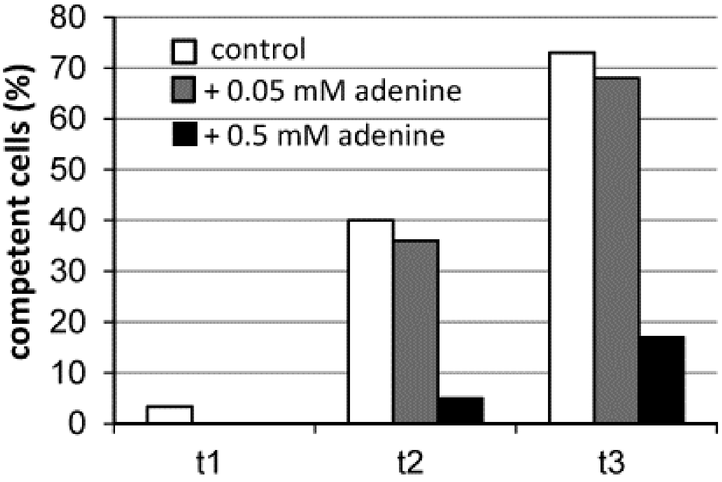

Figure E. Competence development is not influenced by lower concentrations of adenine. Addition of adenine at 0.05 mM (6.8 µg/ml), which were sufficient for purine complementation in minimal medium (see Fig S3), had no effect on competence development. t1, OD_{600nm} of 0.42 ± 0.04; t2, OD_{600nm} of 0.61 ± 0.04; t3, OD_{600nm} of 0.85 ± 0.07.

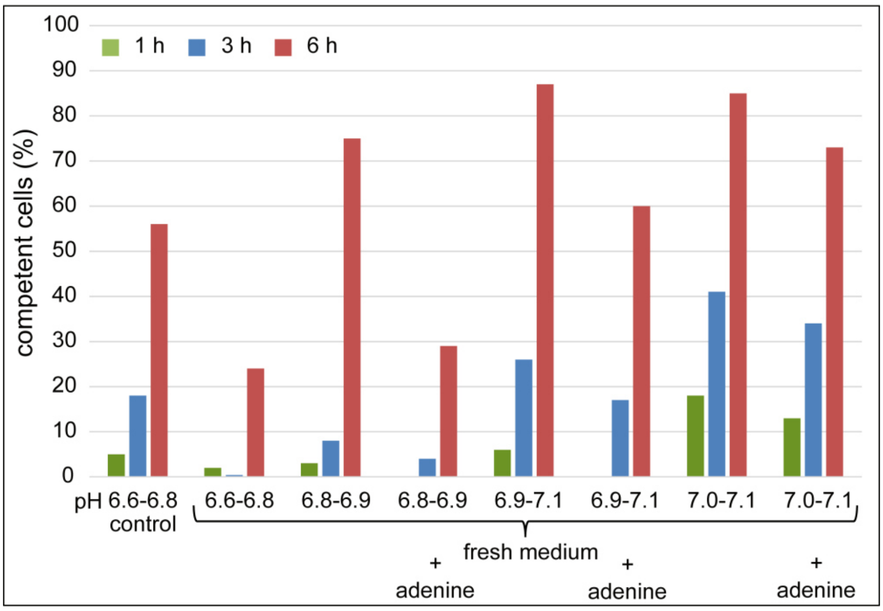

Figure F. pH is a prerequisite of competence development and fine-tuned by the degree of oxidative stress. BB-FBS was titrated to distinct initial pH values (pH 6.6, 6.8, 6.9 and 7.0 after equilibration of CO_2 under microaerobic conditions). A cell suspension of *H. pylori* N6 was grown overnight to t0 (non-competent state) and competence development was followed under microaerobic conditions (control) and after exchange of the supernatant by fresh BB-FBS with distinct initial pH in the absence or presence of 0.5 mM adenine. Note that the overall competence development under microaerobic conditions is slower than under aerobic conditions, confirming the results depicted in Fig. 6. As observed for the control cells, the relative pH increased for about 0.2 units during microaerobic growth over 6 hours (± 0.05 pH units). The increase of the fraction of competent cells in time was higher with increasing initial pH values.
